# Myiasis by *Cordylobia anthropophaga* (Calliphoridae) in rodents from Cape Verde

**DOI:** 10.1007/s11686-022-00576-8

**Published:** 2022-06-10

**Authors:** Ángela Fernández-Alvarez, Santiago Sánchez-Vicente, Carlos Feliu, Basilio Valladares, Jordi Miquel, Joana Alves, Rosario Melero-Alcíbar, Pilar Foronda

**Affiliations:** 1 Instituto Universitario de Enfermedades Tropicales y Salud Pública de Canarias, Universidad de La Laguna, Avda. Astrofísico F. Sánchez, s/n, 38203 La Laguna, Spain; 2 Secció de Parasitologia, Departament de Biologia, Sanitat i Medi Ambient, Facultat de Farmàcia i Ciències de l’Alimentació, Universitat de Barcelona, Avgda. Joan XXIII s/n, 08028 Barcelona, Spain; 3 Center for Infection and Immunity, Mailman School of Public Health, Columbia University, New York, NY USA; 4 IRBio, Facultat de Biologia, Universitat de Barcelona, Avgda. Diagonal 645, 08028 Barcelona, Spain; 5 Departamento Obstetricia y Ginecología, Pediatría, Medicina Preventiva y Salud Pública, Toxicología, Medicina Legal y Forense y Parasitología, Facultad de Farmacia, Universidad de La Laguna, Avda. Astrofísico F. Sánchez, s/n, 38203 La Laguna, Canary Islands Spain; 6 INSP/Ministerio da Saude, Praia, Cabo Verde; 7 Fundación Iberoamericana de Las Ciencias Sociales y La Salud, C/Fuente del Rey, 2, 28023 Madrid, Spain

**Keywords:** *Cordylobia anthropophaga*, Myiasis, *Rattus rattus*, Rodents, Cape Verde

## Abstract

**Purpose:**

The tumbu fly, *Cordylobia anthropophaga* (Diptera: Calliphoridae), is widely distributed in continental tropical and subtropical Africa, being the most common cause of furuncular myiasis in Sub-Saharan Africa. The aim of the present work was to analyze the role of rodents as possible reservoirs of *C. anthropophaga* in Cape Verde, considering the zoonotic character of this fly species.

**Materials and Methods:**

A total of 150 peridomestic rodents were studied in Santiago island. For the obtained larvae, morphological and molecular characters were analyzed.

**Results:**

*Cordylobia anthropophaga* was found in 6.4% of the peridomestic *Rattus rattus* analyzed. The present work unveils the presence of *C. anthropophaga* in rodents of the African archipelago of Cape Verde, introduced probably with West African humans and/or animals.

**Conclusion:**

The presence in peridomestic animals, and the wide range of species that this fly can affect, entails a zoonotic risk of myiasis by tumbu fly.

**Supplementary Information:**

The online version contains supplementary material available at 10.1007/s11686-022-00576-8.

## Introduction

Myiasis is the infestation of humans or vertebrate animals body tissues by the larvae of species of the order Diptera [1]. This pathology caused by diverse species of two-winged flies is globally distributed and is responsible for serious economic losses to the livestock industry worldwide [2]. Humans may also be infected, mainly those living in poor socio-economic countries of tropical and subtropical areas [3]. Five dipteran families are considered predominantly responsible of obligatory and facultative myiasis in humans and mammals: Muscidae, Fannidae, Oestridae, Sarcophagidae and Calliphoridae [3].

*Cordylobia anthropophaga* (Blanchard 1871) (Diptera: Calliphoridae) stands out within the family Calliphoridae as the most common cause of clinical furuncular myiasis in Sub-Saharan Africa [4, 5]. Commonly known as “tumbu fly”, this species has been reported to cause endemic myiasis in Western Africa for more than 130 years [6]. In this study, the presence of *C. anthropophaga* parasitizing peridomestic rodents is reported for the first time in Cape Verde.

## Materials and Methods

As a part of the Macaronesian ecoregion, the archipelago of Cape Verde is located in the North Atlantic Ocean between latitudes 14° 23′-17° 12′ N, and longitudes 22° 40′-25° 22′ W. Situated 455 km off the Western African coast, the archipelago comprises a total of ten volcanic islands and five islets, divided into two major groups according to the position facing northeast winds: Barlovento and Sotavento. Due to its geographical location, in front of the Sahel Belt, the climate of Cape Verde is mainly arid and semi-arid with scarce rainfall and an average annual temperature of 25 °C. The rainy season is normally between July and October with some brief but heavy downpour periods.

**Fig. 1:**
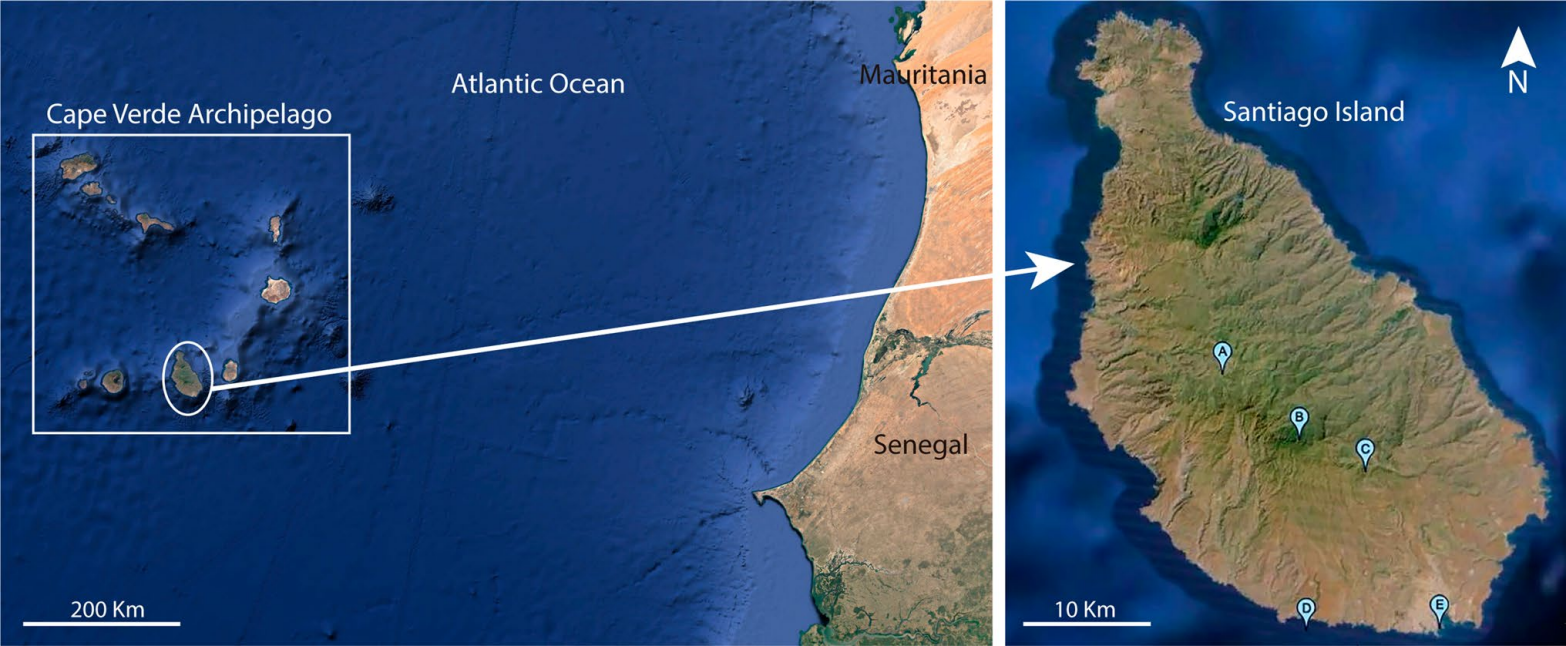
Rodent sampling locations in the island of Santiago, Cape Verde. **A** Assomada, **B** São Jorge dos Orgãos, **C** São Domingos, **D** Cidade Velha, **E** Praia

Two trapping campaigns were conducted on Santiago island in 2012 during the rainy season (July) and dry season (December). Surveyed locations included Assomada (15° 5′ 45.6″ N, 23° 40′ 1.2″ W), São Jorge dos Orgãos (15° 3′ 0″ N, 23° 36′ 39.6″ W), São Domingos (15° 1′ 40.8″ N, 23° 33′ 46.8″ W), Cidade Velha (14° 54′ 57.6″ N, 23° 36′ 21.6″ W), and Praia (14° 55′ 4.8″ N, 23° 30′ 32.4″ W) (Fig. 1). The study areas in the three former localities covered rural habitats mainly characterized by dry forests dominated by acacia and euphorbia species. In Cidade Velha, the study area was located in a fruit tree plantation, and in Praia traps were placed in a water treatment plant situated within the city and surrounded by xerophilic vegetation.

Each locality was sampled using 200 live traps (Sherman and Firobind) during 1–3 consecutive nights. Captured rodents were killed by CO_2_ inhalation. All the animal procedures were performed according to the principles of animal welfare in experimental science. Parasites recovered were preserved in 70% ethanol for morphological characterization and in 100% ethanol for molecular study.

For light microscopic observations, some specimens were treated with KOH (1 h at 60 °C) to clariphy the larvae. They were then rinsed in Milli-Q water (Millipore Gradient A10) and, finally, slides were prepared with Hoyer mounting medium and observed under the light microscope.

For the scanning electron microscope study, other specimens were fixed in cold 2.5% glutaraldehyde in a 0.1 M sodium cacodylate buffer at pH 7.4. They were then postfixed in 1% osmium tetroxide, dehydrated through an ethanol series of increasing concentration, and critical point dried with carbon dioxide in a Polaron CPD 7501. Finally, specimens were mounted on stubs with an adhesive tape and colloidal silver, sputter coated with gold in a Fisons Instrument SC 510, and examined using a Zeiss DSM 940A scanning electron microscope at 15 kV.

Total genomic DNA was isolated from approximately 1/3 portion of the larvae using the FastDNA (BIO 101^®^ Systems) kit, following the manufacturer’s instructions. PCR amplifications were carried out to amplify a fragment of the 5′ region of the 28S rRNA gene, following Stevens and Wall [7]. PCR amplicons were purified with UltraClean PCR Clean-up Kit (MO BIO Laboratories, Inc.) following the manufacturer indications, and sequenced in SEGAI (University of La Laguna sequencing services, La Laguna, Tenerife, Spain). The sequences were analyzed with software MEGA X (Molecular Evolutionary Genetic Analysis) [8] to check the quality of the sequences, and minor corrections, to increase the aligned sequence similarity and improve the inferences on any positional homology, were then made by hand. A BLAST search [9] was carried out to elucidate the homologies of the nucleotide sequences obtained with the sequences previously published in the GenBank database.

## Results

A total of 150 peridomestic rodents, 72 *Mus musculus domesticus* and 78 *Rattus rattus,* were captured at the 5 sampling localities. Twenty-four *R*. *rattus* were captured at Assomada, 2 *M*. *m. domesticus* and 3 *R*. *rattus* at São Jorge dos Orgãos, 59 *M. m. domesticus* and 44 *R*. *rattus* at São Domingos, 2 *M. m. domesticus* at Cidade Velha, and 9 *M*. *m. domesticus* and 9 *R*. *rattus* were trapped at Praia.

During the examination of the animals, several specimens of *R*. *rattus* showed a significant swelling in the front or backlegs. After dissection of the swelling, a purulent exudate accompanied by the larva of *C*. *anthropophaga* was observed. The larvae of *C. anthropophaga* were found in five *R. rattus* (6.4%), four of them collected in São Domingos and one in São Jorge dos Orgãos (Table 1).They were found in the fore-limbs of four of the rats, and in the back of the other one (see video, Supplementary Information). Five more rats presented lesions compatible with wounds caused by myiasis in the fore-limbs, the hind-limbs and in the back, four of them had been collected in São Domingos and the other one in Assomada. Myiasis was not detected in *M*. *m. domesticus*.

**Table 1:** Prevalence of *Cordylobia anthropophaga* larvae in rodents from Santiago (Cape Verde)

Locality	*Mus musculus domesticus* +/n (*P*%)	*Rattus rattus* +/n (*P*%)
Assomada	–	0/22 (0)
São Jorge Dos Orgãos	0/2 (0)	1/3 (33.3)
São Domingos	0/59 (0)	4/44 (9)
Cidade Velha	0/2 (0)	–
Praia	0/9 (0)	0/9 (0)
Total	0/72 (0)	5/78 (6.4)

+  number of animals with *Cordylobia anthropophaga* larvae, *n* number of animals analysed, *P*(%) prevalence

**Fig. 2:**
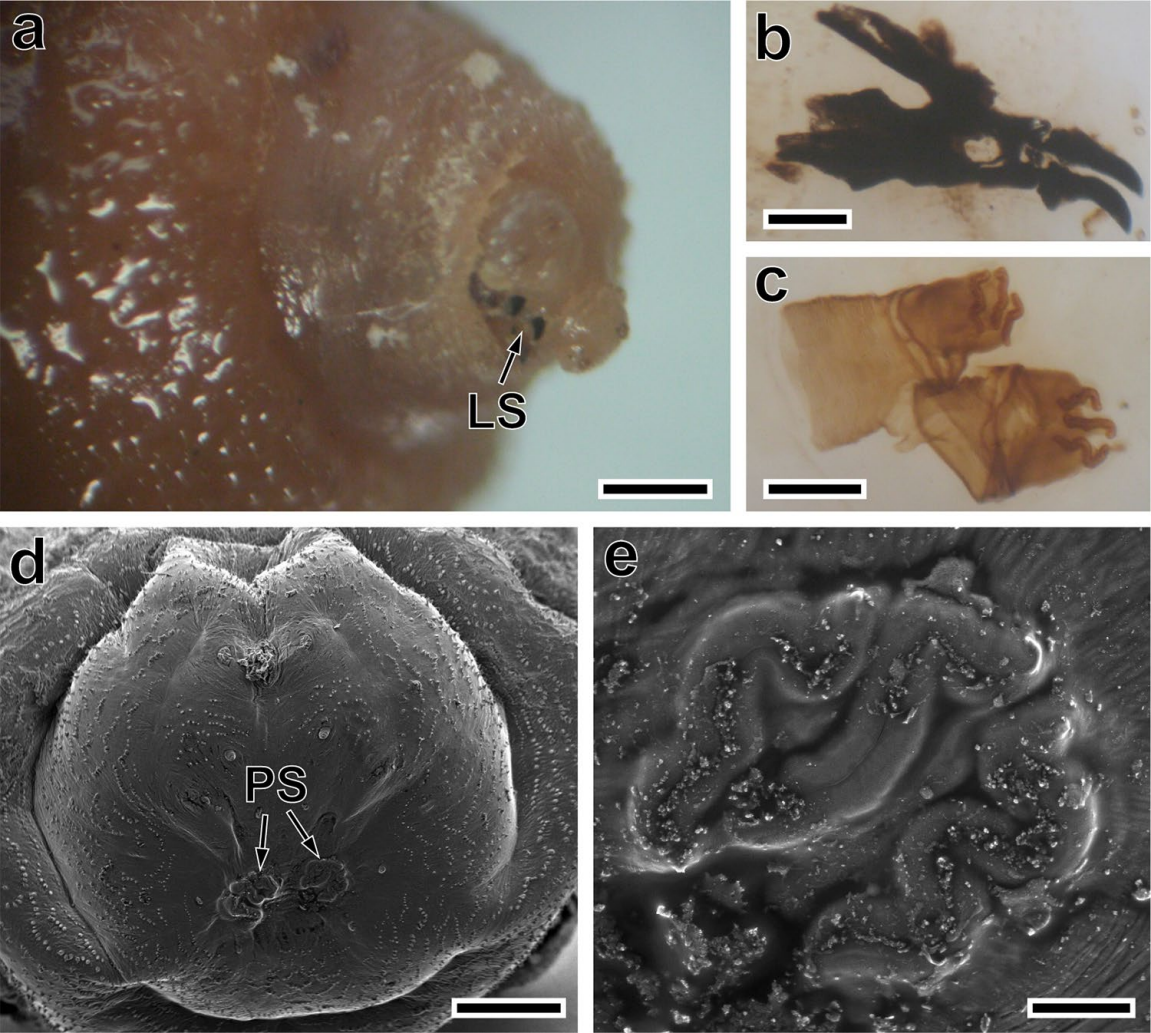
Third larval stage of *Cordylobia anthropophaga*, light and scanning electron microscopy. **A** Anterior tip showing the two labial sclerites (LS). **B** Cephalo-pharyngeal skeleton. **C** Anterior spiracles. **D** Posterior view showing the posterior spiracles (PS). **e** Posterior spiracle. Scale bars: a, d = 500 μm; b = 100 µm; c, e = 50 µm

Larvae were identified as the third larval stage of *C*. *anthropophaga* using standard identification keys, based on their morphological characteristics, including the presence of three slightly serpentine slits on the posterior spiracular plates of the larva [1, 10] (Fig. 2).

A 331bp fragment of the 5′ region of the 28S rRNA gene was sequenced and submitted to the GenBank database under the accession number MT464463. It had 100% of identity with *C. anthropophaga* obtained from Nigeria (F806640.1), Cameroon (AJ551432.1) and Uganda (KM506761.1).

## Discussion

Up to 40 dipteran species have been reported as causative agents of human myiasis worldwide [3]. Among them, *C. anthropophaga* and *Dermatobia hominis* (Linnaeus, 1781) (Diptera: Oestridae) are the most common species detected causing human furuncular myiasis in Africa and in Central and South America, respectively [5, 11]. Another species within the genus *Cordylobia*, *Cordylobia rodhaini* Gedoelst, 1910 (Diptera: Calliphoridae), also causes furuncular myiasis in humans, but its distribution is restricted to the rainforest areas of tropical Africa [12].

Although the archipelago of Cape Verde has a great biodiversity, the terrestrial mammal richness is extremely low. Excluding feral cats and dogs, goats and other domesticated livestock, five non-native terrestrial mammals were introduced by humans throughout the colonial history of Cape Verde: the green monkey *Chlorocebus sabaeus*, the European rabbit *Oryctolagus cuniculus*, the house mouse *M. musculus*, the brown rat *Rattus norvegicus* and the black rat *R. rattus* [13, 14]. The colonization process of the Cape Verde islands began in the fifteenth century when the first Portuguese settlers moved into the archipelago around 1460. Shortly thereafter slaves coming from the West African Coast were used for working in the settler's estates in the Cape Verde islands, maintaining a constant transport flow from the African mainland to the archipelago for nearly three centuries [15]. Therefore, the presence of *C*. *anthropophaga* in Santiago island may be explained by the arrival to the archipelago of parasitized humans, domesticated animals or rats from the West African coast.

A wide variety of animals may be involved in the life cycle of the tumbu fly, being wild rats the main hosts [3]. In Cape Verde, with the present work, the role of these rodents in the life cycle of *C. anthropophaga* was confirmed, and concretely of *R. rattus.* Mice were also analyzed and, although previous studies have found larvae in this host [3], in this case they were not detected. Future studies should be carried out to analyze also *R. norvegicus*, the other rodent species present in Cape Verde that was not collected in this survey.

Moreover, taking into account that dogs are one of the main targets of *C*. *anthropophaga* among the larger animals throughout Sub-Saharan Africa [16–20], the analyses of this host in Cape Verde should be also develop.

Although there is a gap in the data of distribution and habitat choice of *R*. *rattus* in Cape Verde [13], this work pinpointed the black rat mostly in those biotopes characterized by the presence of abundant vegetation and nearby to human settlements. These conditions allow *R*. *rattus* to interact with other domestic and peridomestic animals, as well as with human, what increase the risk of transmission of *C*. *anthropophaga.*

The presence of *C*. *anthropophaga* in peridomestic animals in Santiago island and the wide range of species that this fly can affect, entail an evident risk to public health of myiasis by tumbu fly considering its zoonotic potential, and that almost 53% of the human population of Cape Verde live on this island. For a better understanding of the epidemiology of *C. anthropophaga* in Cape Verde, future studies should be carried out in hospitals and medical centers for human cases, and in veterinary clinics for myiasis in dogs.

## Supplementary Information

Below is the link to the electronic supplementary material.

Video SI Extraction of a *Cordylobia anthropophaga* larva from a *Rattus rattus*
